# Health-related social needs information in the emergency department: clinician and patient perspectives on availability and use

**DOI:** 10.1186/s12873-024-00959-2

**Published:** 2024-03-18

**Authors:** Olena Mazurenko, Adam T Hirsh, Christopher A Harle, Cassidy McNamee, Joshua R Vest

**Affiliations:** 1 Department of Health Policy & Management, Indiana University Richard M. Fairbanks School of Public Health, Indianapolis, IN USA; 2 Department of Psychology, School of Science, Indiana University– Indianapolis, Indianapolis, IN USA; 3 Center for Biomedical Informatics, Regenstrief Institute, Indianapolis, IN USA

**Keywords:** Health-related social needs, Emergency department, Clinician, Staff, Patient

## Abstract

**Background:**

Patient health-related social needs (HRSN) complicate care and drive poor outcomes in emergency department (ED) settings. This study sought to understand what HRSN information is available to ED physicians and staff, and how HRSN-related clinical actions may or may not align with patient expectations.

**Methods:**

We conducted a qualitative study using in-depth semi-structured interviews guided by HRSN literature, the 5 Rights of Clinical Decision Support (CDS) framework, and the Contextual Information Model. We asked ED providers, ED staff, and ED patients from one health system in the mid-Western United Stated about HRSN information *availability* during an ED encounter, HRSN data *collection*, and HRSN data *use*. Interviews were recorded, transcribed, and analyzed using modified thematic approach.

**Results:**

We conducted 24 interviews (8 per group: ED providers, ED staff, and ED patients) from December 2022 to May 2023. We identified three themes: (1) *Availability*: ED providers and staff reported that HRSNs information is inconsistently available. The availability of HRSN data is influenced by patient willingness to disclose it during an encounter. (2) *Collection*: ED providers and staff preferred and predominantly utilized direct conversation with patients to collect HRSNs, despite other methods being available to them (e.g., chart review, screening questionnaires). Patients’ disclosure preferences were based on modality and team member. (3) *Use*: Patients wanted to be connected to relevant resources to address their HRSNs. Providers and staff altered clinical care to account for or accommodate HRSNs. System-level challenges (e.g., limited resources) limited provider and staff ability to address patients HRSNs.

**Conclusions:**

In the ED, HRSNs information was inconsistently available, collected, or disclosed. Patients and ED providers and staff differed in their perspectives on how HSRNs should be collected and acted upon. Accounting for such difference in clinical and administrative decisions will be critical for patient acceptance and effective usage of HSRN information.

**Supplementary Information:**

The online version contains supplementary material available at 10.1186/s12873-024-00959-2.

## Background

In the emergency department (ED) setting, patients’ health-related social needs (HRSN) complicate care and drive poor outcomes. Issues such as transportation barriers, housing instability, food insecurity, and financial strain, can divert provider attention away from resolving illnesses, inhibit patient treatment adherence, and impede access to follow-up care [1, 2]. ED providers’ challenges in addressing patients’ HRSNs while treating their clinical condition create stress and contribute to burnout [1, 2]. Additionally, HRSNs drive repeat ED visits [3], longer total visit time in the ED [4], more healthcare utilization [5, 6], and higher ED costs [7]. Importantly, ED providers frequently encounter patients with HRSNs; estimates suggest that anywhere from one-third to two-thirds of patients presenting to the ED have at least one HRSN [8–10]. Given the importance of HRSNs, federal agencies, provider organizations, and health system experts advocate for the better collection and usage of patient HRSN information in the ED [11].

Patients are generally open to healthcare organizations’ screening for HRSN information [12], and ED providers and staff recognize the relevance and importance of HRSN to care delivery and patient health [12, 13]. However, introducing HRSN data collection and screening in EDs can be challenging. For example, ED providers and staff express concerns over practicalities like which clinical team member should collect HRSN information while delivering medical care in a complex, time-constrained environment [8, 14, 15]. Also relevant is the fact that HRSN information in the ED may be collected and documented in multiple ways. For example, HRSN may be identified through conversations between patients and different members of the healthcare team [16]. Such conversations may be initiated by healthcare staff, or patients may volunteer HRSN information themselves. HRSN may also be collected using screening surveys, although these have demonstrated limited uptake [15]. Regardless, patients may prefer not to report certain HRSNs due to embarrassment [17] or out of concern that their responses might change their treatment or be used inappropriately against them [17–20]. Nevertheless, findings from across multiple care settings indicate patients generally expect providers to take action to help resolve HRSNs when identified [21].

The importance, provider preferences, workflows, multiple means of collecting data, and the growing institutional and policy pressures around HRSNs create a complex dynamic for ED providers, patients, and staff. Moreover, the ED community has noted that obtaining accurate information on HRSNs is a prerequisite to any action [22]. Considering this situation, this study sought to understand what HRSN information is available to ED physicians and staff, and how certain HRSN-related clinical actions align with patient expectations. Knowledge reflective of both provider and patient perspectives will aid in developing actionable interventions for addressing HRSNs that ED patients are more likely to accept, leading to improved outcomes. This study is part of a larger project to improve patient HRSN screenings in the ED by developing clinical decision support (CDS) tools using predictive analytics (AHRQ-1R01HS028008).

## Methods

### Participants & setting

We recruited participants (ED providers, staff, and patients) who practiced at or had received emergency care from a 300 + bed public hospital system in the mid-Western United States. We used a pragmatic sampling strategy to recruit diverse participants across ED providers, clinical staff, and patients. We recruited ED providers (physicians, nurse practitioners (NP)) through presentations to faculty groups and emails. We recruited staff ED members (social workers, case managers, and nurses) using email with organizational leadership assistance. We recruited adult patients (18 years or older) by making phone calls to those recommended by the organization’s community relations department and sending emails to recent ED patients who had agreed to be contacted for research. We reached thematic saturation with 24 participant interviews. Thematic saturation is when no new themes emerge, and the researchers are confident after repeatedly observing similar data instances with well-developed themes. Each participant gave verbal informed consent before the interview. Participants were compensated with a $50 gift card for their time. Our study was approved by the Institutional Review Board at Indiana University.

### Interview guide

We developed an interview guide for this study grounded in the patient HRSNs literature [23, 24], 5 Rights of Clinical Decision Support (CDS) [25] framework, and the Contextual Information Model [26]. The interview guide language was tailored to the collection of HRSNs information (see Appendix). The first half of the interview guide was dedicated to asking participants what HRSNs information is available and how it is gathered and used in clinical care. The patient interviews also probed on how/when/by whom the HRSN information was gathered during and ED encounter. The second half of the interview guide was dedicated to eliciting participant perspectives on the content and design of a CDS tool for addressing HRSNs. The interview’s second half relied heavily on the 5 Rights framework and Contextual Information Model. The 5 Rights framework is widely endorsed and guides CDS design, implementation, and evaluation. It includes important data elements (*right information*); the appropriate member for the information (*right person*); a preferred point in the workflow (*right time*); how the information could be accessed (*right channel*); and how end users receive the information (*right format*). The Contextual Information Model was developed to facilitate the implementation of CDS in clinical care settings and describes individual end users’ perceptions of *fit*, i.e. the level of congruence between the intervention, user, organizational culture, and workflows [27, 28]. We piloted the interview guides for length and content with a nurse practitioner, a social worker, and two patients from our team’s advisory panel.

### Data collection

All participant semi-structured interviews were conducted using an online meeting platform from December 2022 to May 2023. Dr. Mazurenko led the interviews with ED providers (physicians and nurse practitioners). Dr. Vest led the interviews with ED staff (nurses, social workers, and care managers), and Dr. Hirsh led the patient interviews. All interviewers were supported by at least one additional team member for notetaking. Interviews lasted, on average, 33 min. Study team members met repeatedly during the data collection process to assess the emergence of new information. When we had agreed no new themes were being identified, we decided thematic saturation had been reached. We recorded all interviews with consent for transcription purposes. Before each interview, each participant reported age, gender, race, and ethnicity using a web-based survey. ED providers and staff also reported their credentials and years in practice.

### Analyses

Three team members analyzed interview transcripts using a modified thematic analysis approach [29]. We analyzed ED provider and staff transcripts independently from the patient transcripts. This decision was based on two considerations. First, ED providers and staff had day-to-day experience with HRSNs data collection and applications and, therefore, broader experiences than patients. Second, the results of social factor screening approaches are predominately provider-facing; questionnaire results and examination are meant to drive providers’ decisions and actions, not patients’. We began with the provider and staff transcripts. We conducted independent preliminary screenings of three interview transcripts to confirm that interview questions yielded satisfactory responses informing our study questions. Once all interviews were completed, we screened all interview transcripts to create an initial codebook. Three team members tested the codebook reliability by independently applying the codes to three transcripts. We then met and discussed the accuracy and consistency of the codebook and made necessary adjustments. Upon completing the codebook development, we consensus coded each transcript; two team members independently coded the same transcripts and then met to adjudicate any differences through a discussion to reach a consensus [30]. We agreed on a final set of overarching themes and representative quotes. The above process was repeated on the patient transcripts. Once provider and staff transcripts were consensus-coded, we undertook axial coding to identify common, overarching themes. Three team members grouped all codes into overarching themes. We then met to resolve differences and arrive at a final set.

Throughout our analyses, we employed established procedures in the qualitative methods literature to ensure the rigor and validity of our findings [31–33]. These procedures included practicing reflexivity (continually questioning interpretations, seeking answers in the data to verify or challenge interpretations, becoming aware of one’s preconceptions and biases), depth of description (seeking out the rich details of participants’ words), and searching for alternative explanations or interpretations. We conducted the entire analysis using Dedoose qualitative analysis software, version 8.2 (SocioCultural Research Consultants, Los Angeles, CA). As a further check, we presented study findings to members of our advisory panel members for feedback.

## Results

### Participant characteristics

ED providers (Table 1) were evenly divided by gender, mostly white, and had an average of 7.6 years of experience. Staff participants were mostly female, more diverse in terms of race and ethnicity, with an average of 6.1 years of work experience. Most patient participants (Table 1) were female, and half were white. Table 2 contains several quotes illustrating three overarching themes identified in our analyses. The three main themes illustrate what HRSNs information is *available* during an ED encounter and how it is *collected* and *applied* to care.

**Table 1:** Participant demographics

	ED providers (*n* = 8)	ED staff (*n* = 8)	ED patients (*n* = 8)	Total (*n* = 24)
**Gender**				
Female	50.0	87.5	62.5	66.7
Male	50.0	12.5	25.0	29.2
Transgender	0.0	0.0	12.5	4.2
**Race / ethnicity**				
Asian	12.5	0.0	0.0	4.2
Black or African American	0.0	37.5	25.0	20.8
Hispanic	0.0	12.5	25.0	12.5
Multiple / other	25.0	12.5	0.0	12.5
White	62.5	37.5	50.0	50.0
**Age** (mean, sd)	37.8 (7.2)	41.4 (10.9)	47.3 (14.3)	42.1 (11.4)
**Work experience** (mean years, sd)	7.6 (8.2)	6.1 (5.2)	n/a	n/a

**Table 2:** Themes and illustrative quotes about health-related social needs information availability, collection, and use in the emergency department setting

Theme		Description & representative quotes
*Availability*		*Health-related social needs information accessible during emergency department encounters*
	ED provider	“[Asking about HRSNs] is not something that is like normal practice, because it’s hard to do when you don’t have a dedicated screener. So in the hubbub of what is the ED, …if we have to get them home or we have to figure out prescriptions, right, like the if the patient offers up the information, like ‘I can’t afford my prescriptions’ or ‘I don’t have health insurance,’ or ‘I don’t have a way of getting back home’…that’s kind of when those things come out.” (#8)
	ED staff	“On our [EHR] screen we can see if they’re in any financial debt in the hospital system… We can access it, but you have to know where to find it.” (#9)
		Sometimes in the [EHR] you will have like documentation on a patient’s background or history, but a lot of times that leaves you to kind of making judgement on them. Whether they do have insurance. Whether they don’t have insurance. Whether they suffer from homelessness. It’s not always readily available for you.” (#15)
	Patient	“I don’t know that I wanna talk about my finances with somebody in the emergency room.” (#19)
*Collection*		*How information is obtained*
	ED provider	“Mostly, I just ask them.” (#5)
		“Usually it comes up because the patient will say something. Or nursing will come to us and say, ‘Oh, by the way, did you know that he doesn’t have a place to go tonight?’…It’s almost a last-minute, almost like an afterthought, sometimes.” (#2)
	ED staff	“If I know they came in via an ambulance I kind of start talking then like, ‘If you get discharged how are you going to get home?’ (#12)
		“One of our populations, a lot of them are scared to tell us what’s really going on, where they actually live, who they, who’s actually in the home, and what their needs really are because they’re scared of us reporting it to the government. We have to let them know, ‘You know, we’re here to care for your medical,’ and then that’s when they start disclosing. So, that’s a challenge.” (#14)
	Patient	“It feel like it’s more private if I’m writing on the paper.” (#20)
*Use*		*How information should be used in care delivery*
	ED provider	“Addressing some of those is not really within our ability.” (#5)
		“There are a lot of social determinants and each patient has their own, kind of conglomeration of factors…I try to tailor to be patient-specific on what I perceive their needs to be in that moment.” ( #7)
	ED staff	“I think social work does a lot. We get a lot of homeless…Sometimes it’s hard to get them coverage because there’s no mailing address. So then we just use [the ED] as the mailing address for the homeless population. So social work do most of the helping them as far as food, as far as, you know, if they need – on the ED if they need transportation, they’ll call them a cab. And they do things like that.” (#11)
	Patient	“In an ideal world they would connect you with a social worker who would be able to assist you with those things with resources.” (#18)

### Availability: HRSNs information available during ED encounters

ED providers and staff reported that HRSNs information is inconsistently available. Some respondents stated that social needs data is not available at a point of care, whereas others could access only certain types, such as transportation. A physician described it as “*Haphazard*…*It depends on the patient, if we’ve interacted with them before in our system, oftentimes there will be documentation of previous challenges in terms of housing or food insecurity, history of domestic violence…That being said, sometimes our patients come in unidentified initially, so we don’t actually know who they are, or they’re new patients to us, or they’re misregistered and somehow that information doesn’t cross over* (#5).*”* Another physician expressed a similar sentiment: “*In the EHR, we do have a social determinants of health tab. It’s obviously clinically useless at this point, because it’s not something our nurses purposely document in and so if there’s anything in there it’s usually from the outpatient setting* (#6)”. Similarly, one nurse said: “*On our* [EHR] *we can see if they’re in any financial debt in the hospital system…We can access it, but you have to know where to find it* (#9)”. ED clinicians noted that the availability of HRSN data is influenced by patient willingness to disclose it during an encounter. This is how a nurse described it: “*Honestly, in the ED what’s available is what* [patients] *tell us* (#10).

While the interviewed patients were often accepting of the ED clinicians having HSRN information as “*relevant (#22)”* or “*asked all the time* (#18)”, some information was not as willingly disclosed. For example, one patient stated, “*I don’t know that I wanna talk about my finances with somebody in the emergency room* (#19).” Another shared, “*I’m very sensitive about vulnerabilities in my household, because it puts me in a position where they think I can’t take care of my children - they’re gonna take them away* (#17).

### Collection: how HRSNs information is obtained

ED providers and staff collected information on HRSNs using various modalities, including direct conversations with the patient, chart review, or screening questionnaires. Despite using multiple modalities, ED providers and staff preferred and predominantly utilized direct conversation with patients to collect HRSNs. A physician commented: “*The number one way to get at any information is just to ask the patient, it’s the easiest thing to do…most of the patients know the information and, then I don’t have to dig through the chart* (#7)”. This quote indicates that providers and staff did not favor the chart review for collecting HRSNs information. A care coordinator noted: “*We don’t dig into the EHR in the ED* (#11). Furthermore, providers reported a tendency to verbally exchange HRSNs information with their peers rather than documenting in patient notes. One of the physicians commented: “*We make sure that we bring up those issues to the oncoming colleague who will do more of the discharge than I do* (#3).”

ED providers and staff reported that collecting social needs information is not systematic or universal but is prompted by patient characteristics or circumstances. A physician noted: “*I tend to ask those questions when I feel like the social concern butterfly in my head kind of goes off…maybe how a patient looks, how they dress, how they present themselves* (#6).” A nurse echoed this: *“…sometimes, you can kind of just tell that a patient is like malnourished. Then we’ll kind of probe and ask them like, ‘Do you have access to food’ or ‘When was the last time you’ve eaten?’* (#10). At the same time, this nurse – and other providers and staff – noted that patients often volunteer HRSN information: “*Some patients will just tell you they don’t have access to food, they’re hungry…*(#10).”

While ED providers and staff favored direct conversations for gathering HRSN information, patients had disclosure preferences based on modality and team member. For in-person data collection, ED provider attitudes and demeanor were critically important. One patient recounted a recent ED visit: “[The physician] *was kind and professional and didn’t seem like I was a bother to them. And I think that those characteristics do make it easier to open up to somebody. Just going into an emergency room, though, that’s a very difficult situation to answer those questions* (#21).” In contrast, a less reticent patient commented: “*You know which ones care and you know which ones don’t give a crap.* (#23)”. In terms of the *team member*, patients preferred to disclose HRSN information to staff other than ED physicians. One patient stated, “*I wouldn’t talk to the doctor about my social stuff. This is the guy I want to talk to about what’s wrong with me.* (#17).” Another patient observed: “*If I was experiencing any barriers, I wouldn’t necessarily expect the doctors to do much. I would expect the care team, social workers, to be able to provide because they can assess. So I don’t expect them* [i.e. doctors & nurses] *to do anything because that’s not 100% their job. They are diagnosing and prescribing* (#22)”.

This organization (public hospital system in the mid-Western United States) had HRSNs screening questionnaires. However, their usage was inconsistent due to concerns about data quality and the absence of a standardized workflow for data collection. For instance, instead of using standard questionnaires, one nurse reported using “*just questions I’ve been asking my own way, just because I know* [financial strain] *is a problem* (#9).”

Patients differed in their perspectives on written HRSNs screening questionnaires. While some patients were skeptical of written questionaries, others acknowledged the ease of completion and appreciated not having to verbalize sensitive issues. One patient recounted: “*I was feeling horrible. I kind of didn’t want to talk anyway, or actually, I couldn’t talk very well. So, just writing down makes it easier* (#22).” They went on to say: “*…I would say food access and financial insecurity. It kind of makes it hard to talk to someone* [i.e. the physician] *who’s probably making $140,000 a year…It is a little bit harder for people to hear that when you’re food insecurity.”* Another patient noted that a written questionnaire felt more universal and less targeting: “*It’s obvious that other people are getting this same form and so, I feel more comfortable with it knowing that there’s other people that are answering these questions, they’re probably gathering collective information* (#19).”

Patient views varied on for whom HRSN screening was appropriate in the ED. Two patients noted that universal screening of all patients at all ED visits was appropriate, because HRSN were not immediately obvious and were common amongst patients in the system. One patient stated: “*I’m a senior citizen and I feel like they need to ask those questions, ‘cause there’s a lot of seniors who do live alone that need that help. They might need food or things like that…You could be a young person and still not feel safe at home or you could be not have food and need assistance. I think it’s a general question they should ask everyone* (#18).” Similarly, another commented: “*I think that* [the clinical team] *should be trying* [to understand HRSNs] *all of the time. because health problems don’t just pop up out of nowhere. They build up off each other* (#22).” However, other patients had opposite opinions (e.g. “*I don’t think it’s at every visit* (#23))”, stating their preference for more targeted screening based on visit context or for new patients. For example, a patient suggested: “*If there’s somebody that has showed up a couple of times in the ER and has established a pattern of need, I think maybe that might be a good person to reach out to and say, “Hey, do you need any support?*” (#19). Another said information should be collected “…*at least the first time they come as a patient…then they can know the background* (#20).”

### Application: how HRSNs information should be used in care delivery

When HRSNs information is collected, patients have clear expectations that providers or the organization will take action to address these needs. A patient said: “*It would’ve been nice if they had asked the questions or gave me a questionnaire and said, ‘Hey, do you need any help with food? Do you need any help…What can we do to make your life a lot better than having to deal with what you’re going through?*’ (#23). Patients saw no utility in collecting HRSN information without a path to resolution. As one patient pointed out: “*You’re gonna ask me about the need and then cut it off right there without giving me some direction? Not even a resource, you know? ‘Well, there’s places that will help you with gas. Maybe you should check in with one of the community resources,’ but, just to ask me and then not let it – and not go any further, ‘What’s the point?*’ (#19).” Not only do patients expect the HRSN information to be acted upon, but they also want to be consulted on any next steps. As one patient said: “*If I answered a question, I don’t want somebody just to automatically take action on it. I want them to just say ‘We recognize you selected something that you needed. Here’s what we can offer you’* (#24).”

ED providers and staff reported numerous and varied *decisions* regarding patients’ HRSNs. A frequent example is whether and how to adjust medications and treatment plans to better fit patients’ financial situations. For example, a physician reported “*There’s an indication that there’s some financial insecurity…I just tell’em ‘These are the options. We can go for the cheaper option, but I want you to make sure that you’re actually comply with the medication’* (#1).” Providers and staff also reportedly use referrals to connect patients to services to address their HRSNs. One such example was homelessness. A provider explained: “*We call them social admits as opposed to like medical admits…We sometimes do have patients where they’re stable medically, but they don’t have anywhere to go - whether it’s transportation or they’re homeless and we can’t get them to a shelter. We will keep them until we can get them connected with social work, and then have just a better picture of the options for them* (#3).” Additionally, physicians and staff reported providing food, arranging transportation, and providing cash to patients with HRSNs. In addressing food insecurity, a physician reported an organizational-wide effort: “*What’s nice is* [health system’s] *got a system already set up where they’ve got the food pantries and things like that outside. We’ve been giving $30.00 vouchers out for anybody who screens positive for food insecurity* (#8).” Examples of referrals to other in-house or community services included: financial counseling for the uninsured, information on food banks, social workers to arrange transportation, and connections to local shelters for individuals experiencing homelessness. However, referrals were not always easy, as a nurse reported: “*Most people don’t know the resources that are out there. Even if they do, they have to figure out how to get there; they have to be humble enough to go; they have to know that it’s okay for them to get it. They have to understand ‘This is here to help you right now but also in the long run.’…You kinda just have to brainstorm and use as many resources as you can. People don’t know how to use them* (#16).”

Moreover, these actions were bounded by *system challenges*. In general, the nature of ED workflows, HRSN prevalence, and access to care issues facing the patients served by a safety-net organization created broader challenges. A physician described it as: “*In the ER, I’m seeing new patients every single day. All of these people need social work referrals. Most of them need healthcare providers and don’t have access to care, luckily [health system] has a financial counseling and I honestly don’t know how quickly they get followed up, but my hope is that like they get followed up and they get plugged in with some kind of insurance* (#8).” Similarly, because the ED is open 24-hours and 7 days a week, resources are not always available when needed, including social workers, financial counseling, and pharmacy help. These limitations extend to community-based partner organizations. A nurse recounted: “*Yeah, we get a lot of homeless patients. We have some resources for them, like various shelters that they can go to. There’s not very many and…some patients get kicked out of the shelter, so they have literally nowhere else to go* (#10).” The challenges in addressing patients’ HRSNs were a source of stress for providers and staff. A nurse reported: “*I worked last night and I can think of more than half of my patients who expressed some sort of non-healthcare need to me. I think the goal should be to keep people from using the ED for the social issues. The things that suck our soul away are the social things that go along with it* (#13).”

## Discussion

ED providers, staff, and patients reported that HRSNs information was inconsistently available, collected, or disclosed. Collecting data on, and addressing HRSNs is a significant challenge for any organization; it requires new workflows, additional staffing, and data to drive analytics and decision-making [34]. The ED setting is no different in that respect, but the challenges are amplified by the 24-hour nature of care delivery and the patient population. Provider and staff informants noted how, unlike other settings, the ED requires social service staff to be available during non-standard business hours and days. The provision of after-hours and weekend care also conflicted with the availability of community-based organizations that might address HRSNs. In terms of patients, consistent with the literature [8–10], participants suggested HRSNs were common and often needed acute attention. Collectively, our HRSN-focused interviews yielded rich data from key stakeholders that align with broader movements in Emergency Medicine [1, 35, 36], namely that EDs are an underutilized setting to make high-impact gains in patient and population health. Unfortunately, EDs may also be amongst the most difficult settings in which to introduce the resources and processes to address HRSNs.

These findings also indicate the need for a systematic approach to HRSN data collection and response. Inconsistent administration of screening questionnaires or reliance on data collected in other care settings will miss patients in need of services to resolve HRSNs. Additionally, simply relying on patient presentation or visual cues is fraught with assumptions and biases, which can create, maintain, and exacerbate inequities in how HRSNs are collected and addressed [37]. Universal screening is one systematic approach that could address those issues. However, more widespread use of screening tools may not, by itself, rectify the problem. Some patients expressed concerns about sharing HRSNs due to privacy or potential repercussions, which is consistent with other qualitative patient studies [17–20] and a phenomenon that has been observed in analyses of HRSN screening data [38]. Any ED that attempts universal, or even more widespread, screening may have to consider multiple modalities, e.g., offering screening surveys to all patients and following up with verbal screening for nonrespondents. Alternatively, EDs could narrow screening efforts, for example, to high utilizers [4] or for a select set of HRSNs more relevant to emergency care [22], thereby targeting limited resources.

Regarding expected actions in response to HRSNs, patients’ comments most aligned with the concept of “assistance”, that is, the provision of, or connecting to, relevant resources [39]. However, provider and staff perceptions were more aligned with the concept of “adjustment”, defined as alterations to clinical care to account for or accommodate challenges posed by HRSNs [39]. The distinction could have implications for patient perceptions and satisfaction. The manifestation of assistance activities may be more tangible and visible to patients, e.g., vouchers for food, free medications, or taxi fare. In contrast, adjustment manifests as changes to care plans, or selection of alternative medications, or changes in discharge planning. These are critical activities that may be less readily apparent to patients. These are important considerations when deliberating on best practices to maximize patient-centered care. Differences between assistance and adjustment are also important to health system management and policy. Some policy experts argue that health systems should tread carefully before *providing* social services as this can divert scarce resources and have limited success [40].

### Limitations

This study has several strengths. First, the study benefits from the diverse perspectives of key ED stakeholders physicians, staff, and patients, thus developing a comprehensive picture of HRSN issues in the ED. Second, we purposefully interviewed ED staff with different roles to get a wider range of perspectives. Finally, we used rigorous qualitative methods to identify key themes regarding the availability and collection of HRSNs in the ED, as well as the follow-up actions to address them. Nevertheless, our study is not without limitations. First, responses and discussions may be influenced by the characteristics of participants who agreed to be interviewed for this study. Second, ED physicians and staff were all part of a single healthcare system. Thus, our findings may not generalize to other settings.

## Conclusions

In the ED, HRSNs information was inconsistently available, collected, or disclosed. ED patients, providers, and staff differed in their perspectives on how HSRNs should be collected and acted upon. Accounting for such differences in clinical and administrative decisions will be critical for patient acceptance and effective usage of HSRN information.

### Electronic supplementary material

Below is the link to the electronic supplementary material.

**Supplementary Material 1:** Interview guide

## Data Availability

Transcripts of the interviews are not publicly available to preserve individuals’ and organizations’ privacy.
