# Supplementary material for: Health-related social needs information in the emergency department: clinician and patient perspectives on availability and use
Source: BMC Emerg Med. 2024 Mar 18;24:45. doi: 10.1186/s12873-024-00959-2 (PMC10949703; doi:10.1186/s12873-024-00959-2)
Supplement: Supplementary file 1 — Supplementary Material 1: Interview guide [file 12873_2024_959_MOESM1_ESM.docx]

**Health-related social needs information in the emergency department: clinician and patient perspectives on availability and use.**

**Appendix. Interview Guide.**

ED physicians and staff

*Challenges like transportation, financial insecurity, housing issues, and unsafe living environments, appear frequently among ED patients and can complicate care. More and more health care providers are being asked to identify their patients’ social risk factors and help address them. The objective of this study is to understand how this information fits into your work and how to better make use of that type of information.*

1. What information on a patient’s social risk factors is typically available to you?
   *Prompts & follow-ups:* *What don’t you have? What would like to have?*
2. How do you typically get information on your patients’ social risks?
   *Prompts & follow-ups:* *Do you ask patients? Do patients volunteer the information? Do you review the social factor screening questionnaires collected by others? Do you administer those screening questions yourself? Why do you use that method? Do you believe one method results in higher quality or more relevant information?*
3. During a typical patient encounter, do you routinely use information on patients’ social risks decision-making? How so?
   *Prompts: How does it influence your decisions or other clinical actions? Does it initiate new services or treatments? How does it impact patient care? Does it not influence your decisions? Why not?*

*So far, we have been talking about getting information on patients’ risk factors by just asking and questionnaires. I now want to pivot to another method of getting this information: analytics. We could call this automated risk prediction, machine learning, automatic algorithms, or risk scoring. The basic idea is that instead of asking the patient about income, food security, safety, or other social risk factors; we let the computer compile all the data that already exists on the patient and use this data to predict if this patient is at risk for having social. Some of you may have experience with such automated risk scoring in the cases of specific diseases like diabetes or outcomes like the risk of poor post-surgical recovery or other issues like risk or history of opioid abuse. For this discussion, we are talking about that approach as it applies to social factors.*

*Assuming automated risk scoring for social risk factors was available to you in the ED,…*

1. What would you need to know, or have available to you, to be convinced that the risk score was reliable and clinically valid?

*Prompts: If they mentioned an example: You mentioned [INSERT SCORE HERE] as an example you use. Why do you trust that particular risk scoring?*

1. For the sake of this discussion, assume the risk score is reliable and trustworthy, what would be your suggestions to make sure that an automated risk score was of most of use to you and beneficial to patients?

*Prompts & Follow-ups: When should it be available to you in the care process? Which members of the team should have access to it? How could it make decision-making easier? Graphics? Default orders?*

1. What challenges would the Eskenazi ED face in making something like an automated risk score for social risk factors part of routine care? Assume the risk score is reliable and trustworthy.
   *Prompts: Staff trust? Training? Organizational support? Culture? Organizational capacity to respond to social needs? Patient trust?*
2. Opportunity for concluding thoughts and comments.

ED Patients

*Introduction: Being healthy is more than just seeing a doctor. Challenges like transportation, financial concerns, housing issues, unsafe living environments, family responsibilities, and employment problems can create health problems and get in the way of getting care at a hospital. We call these social needs. More and more hospitals are trying to identify patients’ social needs and help address them. This focus group aims to discuss your views on hospital doctors and staff asking about your social needs.*

1. The last time you were in the emergency department, did you talk about your social needs with the physician, nurses, or any other Eskenazi staff member?
   *Follow-ups: What type of information did you share (e.g. housing, finances, transportation, hunger, etc.)?*
2. Please tell me more about how you provided this information. Did the hospital give you a survey to fill out? Or maybe the doctor or nurse asked you a specific set of questions? You might have been sent an email. Maybe you just told it to the doctor or nurse when they were talking to you.
   *Follow-ups: At what point during your visit did this happen? What were the most challenging aspects of providing information this way? What made it easier to share this information? Does it matter how you give them this information (e.g. questionnaire vs talking)? Who did you have these discussions with?*
3. Was there information that you thought might be important for the doctor or nurse to know, but that you did not share?
   *Follow-ups: What type of information were you reluctant/hesitant to share (e.g. finances, legal issues, family situations)? Why didn’t you share it? Would you have been more willing to share it if you had been asked in a different way (e.g., talking with the doctor vs a questionnaire or on a private questionnaire instead of talking about it)?*
4. Tell me about any concerns you have sharing social needs with your doctors and nurses?
   *Prompts: Do you think this is information your treatment team or the hospital needs to know? Why or why not?* *Are there social needs you think the hospital or staff doesn’t need to know about or factors you don’t want them to know about?* *Which ones? Why?*
5. What did you want or expect the doctor / nurse / hospital to do with the information you shared about your social needs?
   *Prompts: Say you had trouble getting to the hospital because your car was in the shop, your friend who drives you couldn’t pick you up, or a family member needed your car. If the hospital asked about your transportation situation, and you told them, what should they do with this information?*
6. Do you think hospitals, doctors, and nurses should be trying to understand the social needs of all their patients all the time? That is should they be asking everyone about their social needs at every visit?
   *Prompts: Or should they only be looking during specific situations? Like this should be the focus of my primary care doctor? Or maybe only when I come to the ED?*

*So far, we have talked about sharing your social needs with your doctor or nurses though just talking with them, them asking questions, or maybe even you filling out a specific questionnaire or survey. We will now talk about a different method that doesn’t involve surveys, questionnaires, or even talking. In this method, we let computers help us to determine what social needs you MIGHT need assistance with.*

*The basic idea is this: instead of asking you about income, food security, safety, or other social risk factors; we take all the information you’ve previously given us as part of your care, like how many times you have been to the doctor, why you had visits, how far away you live, and put this information into a computer program, which will let us determine if you MAY have a current need because of a social factor. Again, this is all information the doctor already has in order to care for you.*

*We experience this a lot in everyday life already. For example, when you go to an online store and search for one item, the store may recommend other things you like. Or if you buy Coca-Cola at the grocery store, the store might start mailing your coupons to purchase other soda products. You watch a TikTok or read a post on Facebook, and you get recommendations to see other similar videos or posts.*

*These examples are all about taking information you have already shared to try to understand you better. Our computer program* in the Emergency Department *would be applying those same ideas to learning about what social needs you MIGHT need help with.*

1. Assume we could use information that way at the hospital or doctor’s office. What effect do you think that would have on the care you get?
   *Prompts & Follow-ups: Why? Do you think it would make the doctor more likely to talk to you about social needs? Should they use it to help you get services to address this need? Should they share it with you?*
2. Assume that hospital or doctor office is using a computer program to figure out if you have social needs. Would you want to know they are doing that?
   *Prompts & Follow-ups: Why would you want (not want) to know?*
3. Previously, we had talked about the different ways you can share information on social needs with you doctors, nurses, and other care providers and some of the challenges and advantages with those ways. For example, we had mentioned [XYZ]. Would using the computer program be better or worse than those other ways?
   *Prompts: What challenges would it fix? What challenges would it introduce or make worse?*
4. Opportunity for concluding thoughts and comments.
